# Regulation of DNA Damage Response and Homologous Recombination Repair by microRNA in Human Cells Exposed to Ionizing Radiation

**DOI:** 10.3390/cancers12071838

**Published:** 2020-07-08

**Authors:** Magdalena Szatkowska, Renata Krupa

**Affiliations:** Laboratory of Medical Genetics, Faculty of Biology and Environmental Protection, University of Lodz, 90-236 Lodz, Poland; magdalena.szatkowska@biol.uni.lodz.pl

**Keywords:** DNA damage response, double-strand DNA breaks, ionizing radiation, microRNA, cancer therapy

## Abstract

Ionizing radiation may be of both artificial and natural origin and causes cellular damage in living organisms. Radioactive isotopes have been used significantly in cancer therapy for many years. The formation of DNA double-strand breaks (DSBs) is the most dangerous effect of ionizing radiation on the cellular level. After irradiation, cells activate a DNA damage response, the molecular path that determines the fate of the cell. As an important element of this, homologous recombination repair is a crucial pathway for the error-free repair of DNA lesions. All components of DNA damage response are regulated by specific microRNAs. MicroRNAs are single-stranded short noncoding RNAs of 20–25 nt in length. They are directly involved in the regulation of gene expression by repressing translation or by cleaving target mRNA. In the present review, we analyze the biological mechanisms by which miRNAs regulate cell response to ionizing radiation-induced double-stranded breaks with an emphasis on DNA repair by homologous recombination, and its main component, the RAD51 recombinase. On the other hand, we discuss the ability of DNA damage response proteins to launch particular miRNA expression and modulate the course of this process. A full understanding of cell response processes to radiation-induced DNA damage will allow us to develop new and more effective methods of ionizing radiation therapy for cancers, and may help to develop methods for preventing the harmful effects of ionizing radiation on healthy organisms.

## 1. Introduction

Ionizing radiation (IR) consists of alpha, beta and neutron particles, as well as X and gamma rays. As a result of ionization, chemical reactions are initiated and they lead to major disorders of a number of cell molecules, including DNA [1,2,3]. Radiation-induced DNA damage initiates the signaling the transduction pathway, known as the DNA damage response (DDR), resulting in the activation of multiple cellular signaling molecules to determine the cell fate, including cell cycle arrest, apoptosis, senescence, autophagy and DNA repair [4,5].

Although radiation therapy has been in use for a long time, it is one of the most effective techniques applied in the eradication of cancerous lesions in humans [6,7,8]. It can be used alone, as well as in combination with surgery, chemotherapy and immunotherapy. X-rays and gamma rays are photons used routinely in radiation therapy to treat various types of cancer. Particle radiation uses electron, proton and neutron beams to fight cancer. Although the majority of cancers are characterized by medium or high sensitivity to radiation therapy, those whose sensitivity is low are still a great challenge for oncologists and require the development of individualized targeted treatment methods [9,10].

In addition to radiosensitivity, the second problem associated with the use of radiation therapy is its high toxicity to normal tissues in the path of the radiation beam or in close proximity. Damage to healthy tissues can also be caused by radiation scattering or by low omnidirectional doses used in modern radiotherapy systems. The next category are lesions distant from the irradiation site, related to the effects of stress at the irradiation site propagated on distant parts of the body by the immune system. The destructive effect of radiation on normal cells is observed directly after radiation in the surrounding tissues and can be shifted in time, even up to several years after eradication of the tumor [9,10,11].

The most lethal forms of DNA damage after ionizing radiation exposure are DNA double-strand breaks DSBs) [4,12]. Taking into account different DNA template requirements and the available set of proteins associated with the changing of the cell cycle phase, there are two distinct DSBs repair pathways, known as canonical non-homologous end joining (C-NHEJ) and homologous recombination (HR). Each of these repair mechanisms is designed for distinct tasks and brings different results for cells [13,14].

When DNA has sustained exogenous damage, the repair proteins are recruited and the cell cycle is stopped at cellular checkpoints. Unrepaired DNA lesions can inhibit transcription and induce programmed cell death to avoid mutation accumulation in the cells carrying DNA damage [15,16] Unrepaired double-strand DNA damage carried out in cancer cells is the expected end result of many therapeutic strategies, including radiotherapy, as they are lethal to the cell. Tumor cells proliferate continuously, while normal cells do so relatively rarely and most of the vital activities fall into the stationary phase. Due to the fact that the DNA repair system by homologous recombination is active only in the cell division phase, when the correct chromatid is needed for effective repair, the search for potential tumor sensitizing targets on radiotherapy in this DNA repair pathway is particularly well-founded. The concept of synthetic lethality involving homologous biologists [17,18,19,20,21,22,23,24]

MicroRNAs (miRNAs) are single-stranded short noncoding RNAs of 20–25 nt in length involved in the regulation of gene expression by repressing translation or cleaving target mRNA. More than 1000 miRNA transcription units have been identified in human genomes.

Several miRNAs, whose expression may change the regulation of DNA damage response (DDR), and HR protein components have been identified after irradiation [25,26,27].

This review discusses the biological mechanism through which miRNAs affect the DNA damage response and homologous recombination repair relating to IR.

## 2. MicroRNA

**Figure 1:**
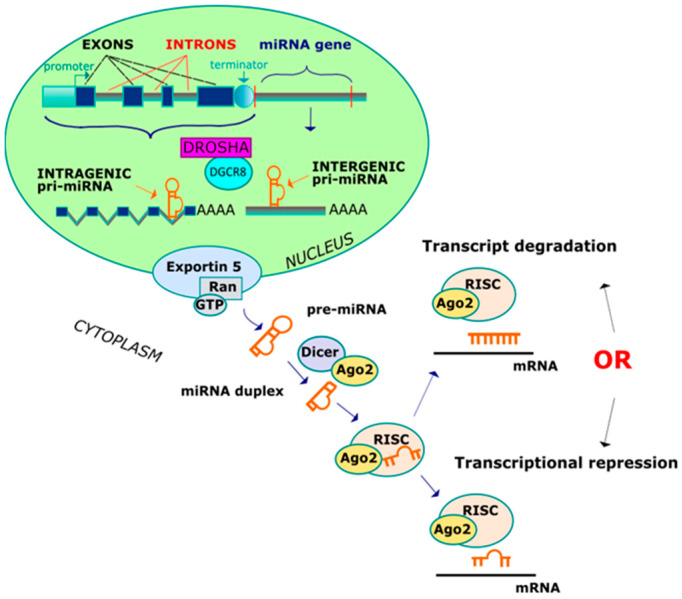
Two ways leading to the creation of miRNA.

The biogenesis of miRNAs comprises several nuclear and subsequent cytoplasmic instances of cleavage that result in the production of mature microRNA. A primary miRNA transcript (pri-miRNA) is synthesized in two different ways, depending of the genomic location (Figure 1) [28,29,30]. The miRNA genes can be classified as either intergenic (miRNA-coding genes contain their own promoter and regulatory sequences) and intragenic (miRNA-coding genes are transcribed with their host genes and from a common promoter region). The intergenic miRNA genes are transcribed in pri-miRNAs and contain a 7-methylguanylate cap at the 5′ end and a poly(A) tail at the 3′ end, which are directly cleaved by Drosha and its cofactor DiGeorge critical region 8 (DGCR8), forming a double-stranded intermediate of ∼70 nucleotide. On the other hand, the intragenic pri-miRNAs are cleaved by the Drosha/DGCR8 complex without affecting the splicing step. Pre-miRNA is subsequently transported by the nucleocytoplasmic transporter factor exportin-5 and ran-GTP, and in the cytoplasm, the enzyme termed DICER (RNase) converts a pre-miRNA into a miRNA duplex intermediate. One strand from a double-stranded miRNA (guide microRNA strand) is loaded onto an Argonaute 2 (AGO2) protein, while the other strand is normally degraded. The AGO2 protein is incorporated into the miRNA guide, generating an RNA-induced silencing complex (RISC) which is guided to the target mRNAs and leads to post-transcriptional gene silencing or mRNA degradation. Imperfect complementarity between miRNA and the 3′ untranslated region (3′UTR) of messenger RNA confers translational repression, whereas if the match is perfect, mRNA undergoes enzymatic cleavage. After IR exposure, significant reductions in either DICER or AGO2 have been noticed, resulting in programmed cell death.

MicroRNA binding sites are located in the 3′ untranslated region (3′UTR) of mRNA [31]. The 3′UTR isoforms can play a different role in gene expression and protein synthesis. An inverse correlation was found between 3′UTR length and mRNA stability, gene expression and the proliferation efficiency of cells. Short isoforms are characteristic of highly proliferative cells, including cancers. Being less liable to degradation, short isoforms are more stable and have a higher transcription and translation ability. Short isoforms contain less microRNA binding sites, which significantly increase the translational potential of mRNA [32,33].

Each alternative 3′UTR isoform has its own set of functional RNA-binding proteins and microRNA binding sites. The binding ability of microRNA depends on the sequence and conformation of 3′UTR, which leads to the fact that any sequence changes that modify the conformation directly—such as mutations or polymorphisms—affect the functionality of 3′UTRs. The protein level is determined by 3′UTRs through the regulation of both mRNA stability and translation efficacy [32,34]. These tasks are based on AU-rich sequences and microRNA binding sequences [32,35,36,37].

The removal of AU-rich elements and miRNA-binding sites (21) from 3′UTRs of proto-oncogenes leads to the development of cancer [37,38]. See other reviews for more details [32,33].

Polymorphisms in microRNA-binding sites in the genes coding for proteins of DNA double-strand break (DSB) repair can influence the risk and prognosis of several types of cancer, including head and neck, colorectal, bladder and oropharynx [39,40,41,42].

Somatic mutations in the 3′UTR sequence do not interfere with the protein sequence. They can interfere with the interaction between the microRNA and its target within the mRNA molecule by directly altering the degree of alignment with the target sequence or indirectly affecting conformation, and thus the availability of microRNA to target sites. A large-scale analysis of 67,159 somatic mutations that can alter the microRNA and mRNA interactions in 21 types of cancer demonstrated the inversed relationship between expression and microRNA–mRNA affinity levels. Functional mutations of 3′UTR microRNA binding sites were more often present in the mitogen-activated protein kinase (MAPK) and WNT signaling pathways, well known to be involved in cancer development [43].

## 3. IR Induced DNA Damage

**Figure 2:**
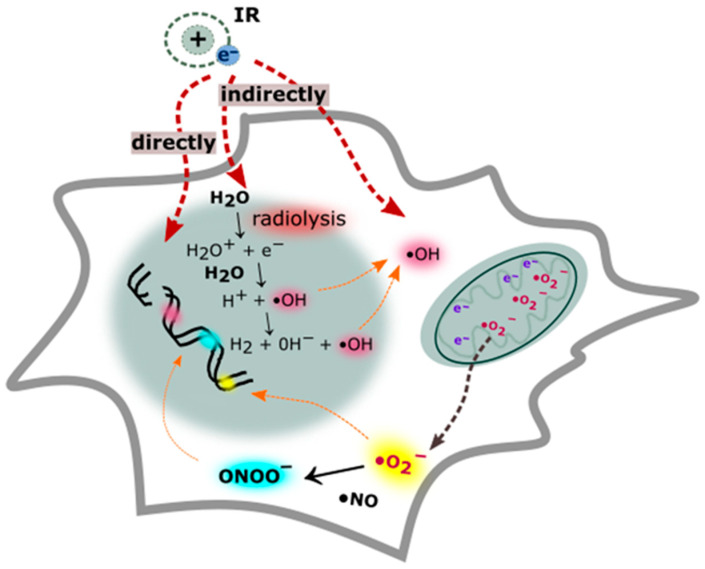
Direct and indirect formation of DNA damage. The absorption of ionizing radiation by living cell hits the DNA molecule directly and disrupts the chemical structure of the DNA double helix. The indirect effect of ionization depends on the radiolysis of cellular water and cellular component disruption. The removal of an electron from water leads to alterations in the nuclear and mitochondrial genome via the overproduction of reactive oxygen species (ROS) and reactive nitrogen species (RNS). During ionizing radiation (IR) exposure, large amounts of nitric oxide (•NO) are generated, which is relatively unreactive and, together with superoxide (O2^•−^), they form a peroxynitrite anion (ONOO^−^)—a powerful oxidant.

Gamma and X rays are the most penetrating radiations and their deposition of energy can directly influence the DNA structure; however, DNA lesions may also be caused indirectly [3,5]. This type of radiation passes through the radiolysis of water contained in the cell and disrupts other organic molecules, which leads to the production of reactive oxygen species (ROS) and reactive nitrogen (RNS) species (Figure 2) [44]. The hydroxyl radical is the most important ROS that interacts with DNA. The hydroxyl radical generates single and double-stranded breaks in DNA by interacting with a sugar molecule in the phosphodiester chain [45,46,47]. It also damages the purine and pyrimidine bases, while purines are damaged with greater efficiency than pyrimidines. The frequently observed DNA base lesions are 8-oxo-7,8-dihydroguanine (8-oxoG), 2,6-diamino-4-hydroxy-5-formamidopyrimidine (Fapy-G), 8-oxo-7,8-dihydro-20-deoxyadenosine (8-oxoA) and 4,6-diamino-5-formamidopyrimidine (Fapy-A) and 5,6-dihydroxy-5,6-dihydrothymine (Thy-Gly). Ionizing radiation exposure to DNA results in apurinic/apyrimidinic sites and more than 100 different lesions [17,48,49].

The genome-wide DNA sequence preference of gamma radiation-induced double-strand DNA break formation was investigated in purified human DNA. C nucleotides—followed by G and T nucleotides—were found to be most prevalent at the cleavage site. A nucleotide was the least prevalent. This means that sequences rich in CG pairs are most exposed to DNA cleavage. The explanation of this phenomenon is conditioned by the fact that the GC base pair forms a wide, major groove in the DNA molecule, which allows the hydroxyl radical to penetrate inside the molecule and interact freely with this base pair. This observation was confirmed by various studies [50,51,52].

Yard et al. [53] conducted a large-scale genetic survival study on 533 genetically annotated human tumor cell lines after exposure to radiation therapy. Cancer cell survival was correlated to somatic copy number alterations. The top 19 genes that were associated with radiation sensitivity when mutated were organized by biological function. Seven of these genes, i.e., *TPR18*, *FLNA19*, *TP53BP1*, *SMG1*, *RANBP9*, *SMARCA4* and *STAG3*, were previously implicated in the DNA damage response [53].

## 4. Double-Strand DNA Break Recognition and Repair

After the introduction of DSB, it is detected rapidly. The DNA damage is quickly detected by poly [ADP-ribose] polymerase 1 (PARP1), which catalyzes the formation of poly (ADP-ribose) chain facilitated attachment of the MRN (MRE11, RAD50, NBS1) complex at the DNA damage site. It is also postulated that both PARP1 and MRN are double-stranded DNA break sensors, which recognize different types of damage [18,19,54,55].

The activation of the DDR signal cascade requires an interaction between the MRN complex and ATM serine/threonine kinase. ATM kinase is the most common intermediate in a number of cellular responses to IR-induced damage [18,56,57]. While the MRN complex is attached to a DNA double-strand break site, the carboxy terminus of the NBS1 protein interacts with ATM and recruits it to the site of DNA damage where its activation takes place. ATM occurs in an inactive form as a homodimer, which is self-activated by phosphorylation after recruitment to the DNA damage site.

ATM undergoes autophosphorylation on Ser 1981, which leads to the breakdown of the inactive dimer into catalytically active monomers [58,59,60]. Moreover, Kozlov et al. confirmed that ATM also undergoes autophosphorylation on Ser367 and Ser1893, thus recruits ATM to broken DNA molecules [58].

The scope of the ATM-mediated in DDR is very wide. Active ATM kinase monomers phosphorylate over 700 protein substrates of this kinase. ATM functions focus on activating DNA repair proteins and cell cycle checkpoint-related factors [58,61,62]. 

Histone H2AX is rapidly phosphorylated at the C-terminal Ser residues (Ser136 and Ser139). The phosphorylation of H2AX on Ser 136 and 139, named γ-H2AX, leads to chromatin modification, which allows other DDR protein components to be recruited. However, upon the recognition of DNA lesions, the deregulation of DDR and other self-repair mechanisms may lead to cell radiosensitivity to radiation therapy. The overexpression of miR-24 and miR-138 targets the histone H2AX transcript at 3′-UTR, which leads to the downregulation of the H2AX histone coding gene and reduces the formation of foci of phosphorylated H2AX following DNA damage [63,64,65,66]. 

C-NHEJ predominates in the G1 phase, whereas it can occur in other cell cycle phases, when an intact sister chromatid is unavailable to guide the accurate HR repair mechanism [67]. Cohesive ends in the DSBs may be simply joined in the C-NHEJ pathway, but if the DNA contains blunt ends, it may result in the deletion or insertion of base pairs. Therefore, the DSBs generated by irradiation require a set of NHEJ factors which ensure rapid repair and maintenance of genome integrity. An alternative form of NHEJ which can be unmasked in the absence of functional C-NHEJ genes are described as alternative end-joining (alt-NHEJ or A-EJ) pathways [68,69].

A-EJ is also described as microhomology-mediated end joining (MMEJ) and is associated with deletions at the repair end junctions. This repair process requires end resection and microhomology sequences that are distant from the DSB. The A-EJ is suppressed by C-NHEJ and HR, although if these standard repair processes fail because a cell is deficient in C-NHEJ crucial proteins, the A-EJ is recruited to repair the damage [69,70].

HR repair is restricted to the S, G2 and M phases to ensure the correct cell divisions. The activity of HR involves resection at the DSB and repair using a DNA homology template, leading to accurate repair, although occasionally it may also contribute to mutation, albeit to a much lesser extent [71,72].

The single strand annealing (SSA) is mainly active in yeast and mediates end joining between interspersed nucleotide repeats. The repair pathway is independent of the cell cycle and is not associated with the requirement that a sister chromatid be present. This is a homology-directed repair which removes DSBs by annealing a DNA segment close to the break with a neighboring homologue, leading to deletion of genetic information between the repeats [73,74].

Unrepaired DNA damage causes cancer cell death by apoptosis, necrosis or mitotic catastrophe. The mode of the cell death depends on the cell type, cell cycle phase, dose of irradiation and cancer environmental properties such as oxygen availability [75,76]. Some double-strand DNA breaks are very difficult to repair. They remain persistent and do not lead to cell death but bring it to a state of senescence [55,77,78]. The senescence-associated secretory phenotype (SASP) is responsible for the induction of inflammatory cytokines, extracellular matrix remodeling and the stimulation of angiogenesis to promote tumor growth and metastasis formation. Some factors, such as those secreted from stroma cells, can reverse the senescence of cancer cells, which is a common cause of cancer recurrence [78,79,80].

Cancer cells are highly heterogeneous. One population of these cells—cancer stem cells (CSCs), characterized by the ability of self-renewal and indicated as the main cause of cancer metastasis—are highly resistant to ionizing radiation [81,82].

The incapability of DDR and DSBs repair pathways is commonly considered to be a cause of carcinogenesis by increasing the mutation rate, which—in consequence—leads to the development of heterogeneous lineages of cancer cells and resistance to chemo-radiotherapy [83,84,85]. On the other hand, targeting some of its components leads to the sensitization of cancer to IR. As indicated, the inhibition of important HR repair components, such as recombinase RAD51, provides promising results in terms of sensitizing cancer cells to radiotherapy [55,86,87,88,89,90,91,92,93].

Repair based on a homology template allows DNA damage to be repaired accurately and the risk of mutagenesis to be reduced. Although HR repair might not be completely error free, it is much less mutagenic than NHEJ [84,94,95,96,97,98]. The NHEJ deficiency is less often observed in context of tumorigenesis than HR [99,100]. Therefore, HR is a good candidate for use in cancer therapy based on the synthetic lethality strategy.

**Figure 3:**
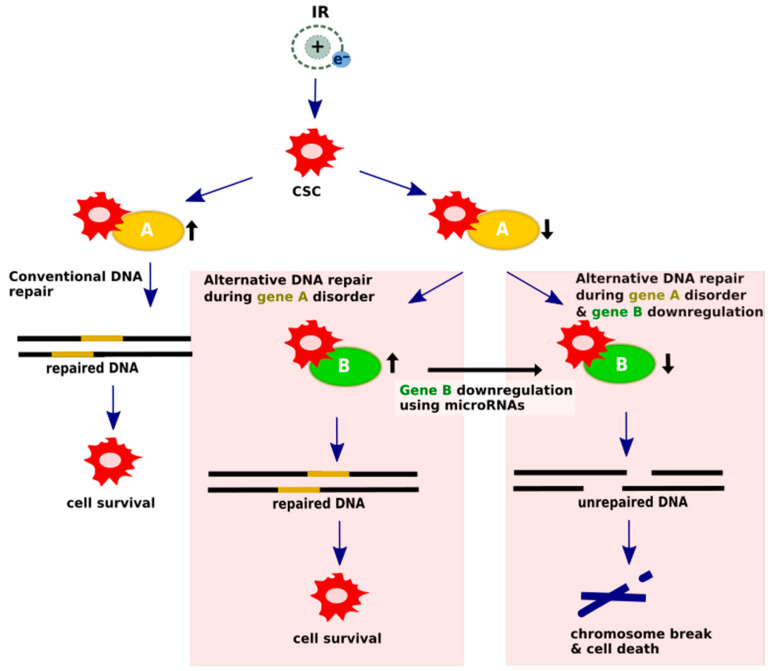
Synthetic lethality mechanism undergoes microRNA regulation. In conventional pathways, DSBs are repaired and cells are able to survive; however, cells with DNA-repair protein deficiencies rely heavily on alternative mechanisms to repair damaged DNA. The concept of alternative DNA repair pathways using microRNA modulation assumes that silencing a crucial gene factor provides chromosome discontinuity and cell death. Upregulated DNA damage repair genes are assigned as black arrows pointing up, while suppressed genes are presented as black arrows pointing down.

The synthetic lethality strategy is dedicated to cells with mutations, polymorphisms or epigenetic changes that cause a loss of function of one of the DNA repair proteins, as a highly redundant DNA repair system uses an alternative or complementary pathway to repair the damage. Through the additional artificial inactivation of the relevant genes of those pathways, DNA repair in pathological cells can be significantly impaired, thus leading to selective death. MicroRNAs are promising candidates for such inactivating agents [101,102]. A scheme of the potential microRNA participation in generating synthetic lethality is shown in Figure 3.

## 5. MicroRNA Regulation of DNA Double-Strand Break Recognition and Homologous Recombination Repair after IR Exposure

**Figure 4:**
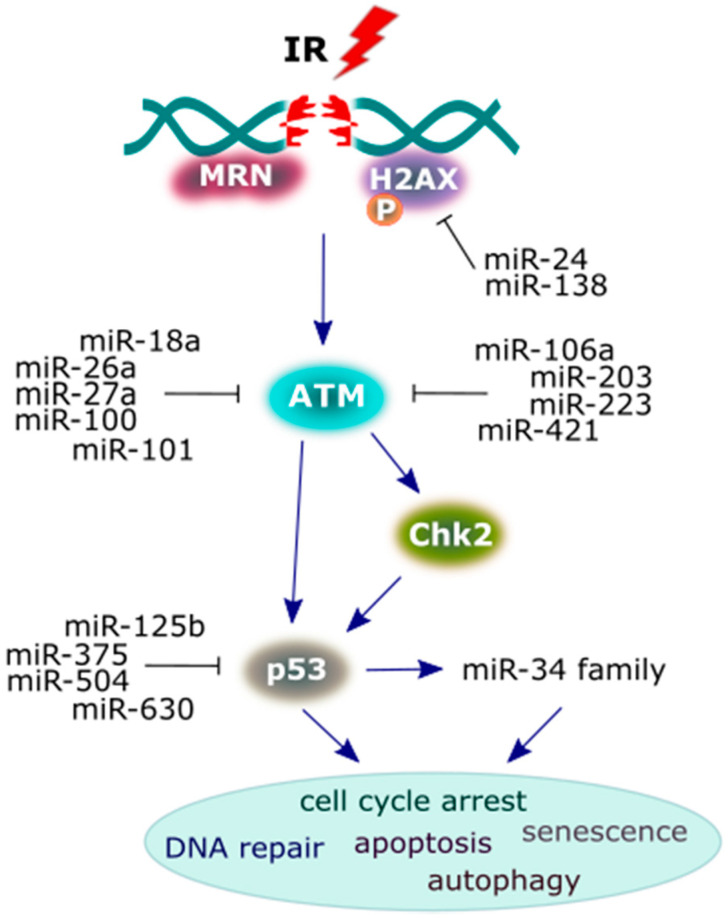
A scheme presenting the major proteins of the DNA damage response (DDR) signaling pathway and the miRNAs that interact with them. Following the induction of DNA double-strand breaks (DSBs) by irradiation, the reaction initiates protein components, such as sensors (MRN/H2AX), transducers (ATM/BRCA1/checkpoint protein 2 (Chk2)) and the effector protein (p53) in order to achieve a cellular response. Multiple miRNAs play a crucial role in the suppression of these DDR proteins in irradiated cancer cells, and some of these proteins may activate miRNAs, leading to a specific cellular response (see the main text for more details).

MiRNAs that regulate several important DDR proteins are demonstrated on Figure 4 and Table 1; Table 2. Several miRNAs that sensitize cells to IR by targeting the 3′-UTR of ATM have been identified in cancer cells. Upregulated miRNAs include the following: miR-18a [103], miR-26a [104], miR-27a [105], miR-100 [106], miR-101 [107], miR-106a [108,109], miR-203 [110], miR-223 [111], and miR-421 [112], leading to the suppression of the ATM gene and formation of its protein product at nuclear foci. Furthermore, miR-421 is upregulated by the proto-oncogene protein, N-Myc, transcription factor that establishes some signaling cascades (miR-421/N-Myc/ATM) that causes cell radiosensitivity [112]. The key downstream target substrate, phosphorylated by ATM, is checkpoint protein 2 (Chk2), mediating the effects of ATM on DNA damage repair mechanisms and other cellular responses that consequently halt the cell cycle [113]. Chk2 subsequently phosphorylates p53 (a tumor suppressor protein), because its activation determines the fate of the cell. Moreover, p53 is also stimulated directly by ATM kinase [114]. The overexpression of miR-125b [115], miR-375 [116], miR-504 [117] and miR-630 [118], leading to the repression of the endogenous level of p53 protein, and the loss of the p53-coding gene function, predisposes the organism to tumor growth.

The tumor suppressor p53 mainly acts as a transcription factor. After IR exposure, p53 undergoes post-translational modification, leading to the induction and/or inhibition of many transcriptionally activate target genes [119,120]. The connection between p53 and miRNAs is also described. MiRNAs are considered to be intrinsic components of the p53 pathway. Among the current data, three miR-34 family members (miR-34a, miR-34b, and miR-34c) have been identified that are transcriptionally activated by the p53 protein. The miR-34 family is encoded at two distinct genomic loci—mir34a and mir34b/c—that contain identical seed sequences. In addition, in mammalian cells, the miR-34 family is often expressed by p53 in response to IR. Due to reductions in the level of miR-34 expression in a variety of tumor cases, as well as associated genomic deletions and promoter hypermethylation, it was confirmed that miR-34 can play a role as a radiosensitizing agent and potential therapeutic target in an anti-miR-34 approach [121,122].

Ionizing radiation induces RAD51 nuclear foci formation and the regulation of HR repair by miRNAs. A growing body of evidence clearly indicates that miRNAs are specifically regulated with regard to IR dose and DNA repair time in response to IR. Moreover, some miRNAs can promote the overexpression of certain HR factors in several cell lines or may lead to the downregulation of another HR protein in other cells [123,124].

**Figure 5:**
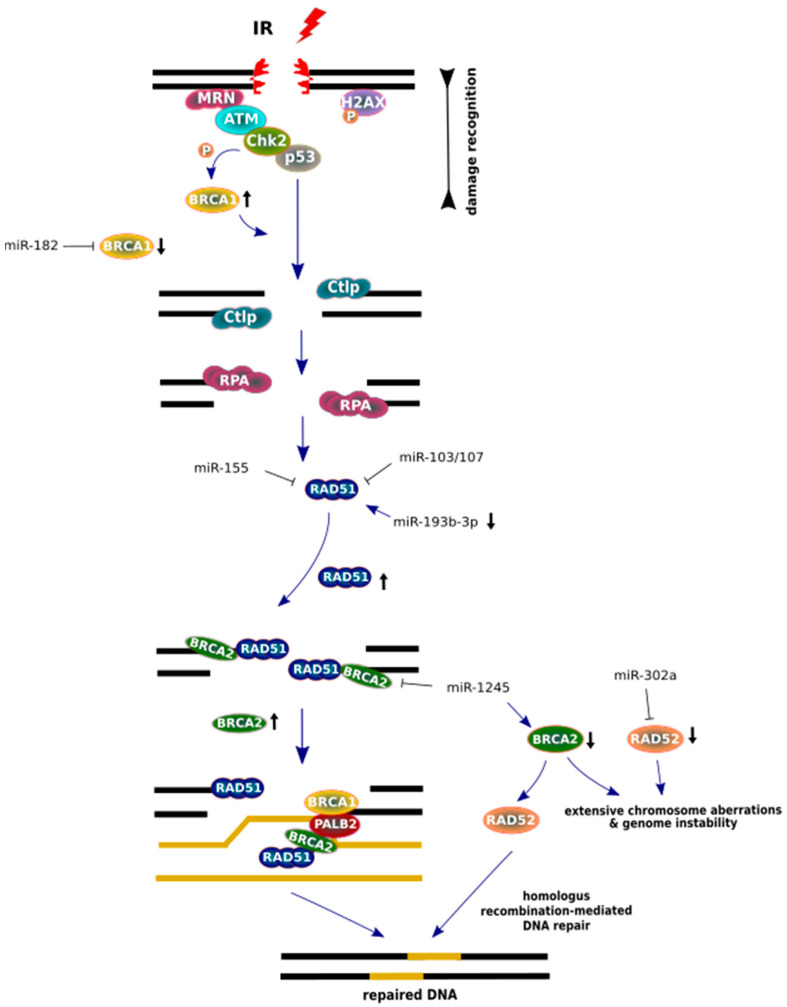
MiRNA regulation of homologous recombination (HR) repair factors after IR. After double strand break (DSB) recognition, BRCA1 (black arrow, pointing up) is phosphorylated by Chk2 kinase and an endonuclease CtIP is delivered for promotion end resection. While, at a later step of HR, BRCA1 recruits PALB2, which mediates promotion of BRCA2 to arrangement on the DNA template. Active BRCA2 (black arrow, pointing up) proteins displaces replication protein A (RPA) in order to load RAD51 onto the DNA. However, low BRCA2 protein levels cause increased levels of RAD52, which promote RAD51 for accurate homologous recombination (HR) repair in BRCA2-deficient cells. The lack of BRCA2 and RAD52 (black arrow, pointing down) causes the most severe genome defects. Major components of the HR pathway are arranged in a scheme based on the aspect of the miRNA regulation post-IR.

The MRN complex is responsible for the initial recognition of DSBs that generate single-stranded DNA and G2/M checkpoint arrest for HR repair. During HR processes in eukaryotic cells, RAD51 participates in the majority of the repair (Figure 5) [125,126,127]. As a central player in the HR mechanism, RAD51 is overexpressed in tumor cells, because malignant cancer cells often bear p53 mutations in tandem with a low-level DNA damage sensitivity caused by chemo- and radiotherapy treatment [120,128,129,130,131].

Several miRNAs have been described as important regulators of HR proteins, which change their expression level after irradiation treatment. HR repair activation is mediated through the ATM/Chk2/p53 signaling pathway and requires many protein factors (BRCA1, BRCA2, PALB2) and RAD51 paralogs (RAD51B, RAD51C, RAD51D, XRCC2, and XRCC3) for the proper post-translational modification of RAD51 and accurate repair after irradiation [142,143]. Manipulation of HR mechanism by using miRNAs regulation leads to a clear increase in radiosensitivity or IR-resistance promotion in many cancer cell lines [103,104,108,144,145]. For example, irradiation may promote RAD51 expression by downregulating miR-193b-3p in hepatocytes, whereas than the miRNAs (miR-1255b, miR-148b* and miR-193b*) are inhibited, the increased expression of BRCA1, BRCA2 and RAD51 is detected [123,146].

It is important to note that IR induces RAD51 nuclear foci formation, which is cell cycle-dependent [153]. The regulation of HR proteins by specific miRNAs may promote genomic instability after IR exposure. For example, irradiation may promote RAD51 coding gene expression by downregulating miR-193b-3p in hepatocytes, whereas if miRNAs, including miR-103/107 and miR-155, are inhibited, then an increased expression of RAD51 is detected [26,133,146,154].

BRCA1, a tumor-suppressing protein, epigenetically represses miR-155 in lung cancer cell lines, and the inhibition of miR-155 may have anti-cancer potential in sensitizing hypoxic lung cancer [144,155]. On the other hand, it is considered that miRNAs such as miR-103 and miR-107 consistently reduce IR-induced RAD51 foci formation in endometrial and bone cancer cell lines [156]. As a result of radiation, the transcripts of genes coding BRCA1 are targeted by miR-182 overexpression, which leads to gene silencing [152]. BRCA1 interacts with numerous molecules and its deficiency is often related to breast cancer development. However, a clinical prognostic factor, BRCA2, is also co-localized with RAD51 and the RAD51/PALB2/BRCA1 complex during HR repair. In breast cancer cells, the upregulation of miR-1245 is noted after IR treatment. The inhibition of miR-1245 may enhance BRCA2 expression and RAD51 nuclear foci formation [157,158,159,160,161]. Feng et al. showed that RAD52 plays a role as an alternative factor that is essential for the survival of BRCA2-deficient cells, while *RAD52* deletion may reduce cancer progression [162]. MiR-302 represses *RAD52* transcripts in breast cancer cells, providing to radioresistance and allow cancer cells to survive [163].

## 6. Conclusions

Short non-coding RNAs, miRNAs, regulate many cellular factors, which participate in DDR and HR repair mechanisms after irradiation.

Following irradiation, miRNAs are involved in regulating HR repair in several ways. HR transcripts can be mediated by downregulation or upregulation. On account of IR exposure, some HR proteins may promote the expression of miRNAs, which regulate another protein related to the HR mechanism.

MiRNA regulators may play a role as prognostic factors in cancers.

The sensitization of cancer cells to ionizing radiation by the deregulation of DNA damage response proteins can be crucial for the elevation of effective cancer therapy.

## Figures and Tables

**Table 1:** MicroRNAs that affect DNA damage response and homologous recombination repair downregulated after irradiation.

miRNAs	Target Proteins	Studied Material and IR Dose	Predicted Consequences for Cells	References
miR-155, miR-375	RAD51 p53	Thirty-two male FVB/NJ mice, 12 weeks old/liver tissue Dose: 28 mGy	-	[116,132,133]
miR-34a	c-Myc	Human colorectal cancer cells HCT116 p53-/- Doses: 2; 4; 8 Gy	MiR-34a is a critical mediator of p53 function.	[134,135]
	c-Myc	Human non-small-cell lung carcinoma (NSCLC) H460 Doses: 1; 2; 5 Gy	Senescence-promoting effect.	
miR-24, miR-103 & miR-107, miR-106a, miR-155	H2AX, RAD51 ATM RAD51	Human B lymphoblastic cell line IM9 Dose:1 Gy	MiR-34a may be involved in the cell cycle response and apoptosis pathway in association with p53.	[26,64,108,133,136]
miR-24, miR-26b, miR-125b, miR-100	H2AX ATM p53 ATM	Normal human fibroblasts AG01522 Dose: 10 Gy	Regulation of cellular response following irradiation.	[64,104,106,137,138]
miR-203	ATM	Normal thyroid cells Doses: 1; 10 Gy	MiR-203 dysregulation is associated with radiation exposure and may be unique for thyroid cells.	[139,140]
miR-504	p53	Human head and neck epithelial malignancy, nasopharyngeal carcinoma (NPC), radio-resistant cell lines CNE2-IR and HK1-IR Doses: 2; 4; 6 Gy	Induction of radioresistance by down-regulating the expression of NRF1 and disturbing mitochondrial respiratory function.	[117,141]

**Table 2:** MicroRNAs that affect DNA damage response and homologous recombination repair upregulated after irradiation.

miRNAs	Target Proteins	Studied Material and IR Dose	Predicted Consequences for Cells	References
miR-34a miR-421	c-Myc ATM	Thirty-two male FVB/NJ mice, 12 weeks old / heart tissue. Dose: 28 mGy	The expression of inflammation-related miR-155 changed by low dose irradiation.	[112,132,134]
miR-375	p53	Thirty-two FVB/NJ mice, 12 weeks old testis tissue. Dose: 28 mGy		
miR-34a	c-Myc	Human colorectal cancer cells HCT116 p53 +/+. Dose: 2; 4; 8 Gy	MiR-34a is a critical mediator of p53 function.	[134,135,147]
		Human non-small cell lung carcinoma (NSCLC) A549. Dose: 2; 5; 10 Gy	Senescence promoting effect.	
		Human breast cell line / non-cancerous MCF-10A and cancerous MCF 7. Dose: 5 Gy for MCF-10A additional doses: 3; 12; 48 mGy	Mir-34a is up-regulated in p53 positive cancer and normal cell. MiR-34a might be involved in breast cell responses to low dose radiation.	
miR-18a	ATM	Patients from radiosensitive group /cervical cancer cells. Dose: 8 Gy	Attenuation of DNA DSB repair and re-sensitization of cancer cells to radiotherapy by promoting apoptosis.	[103,148]
miR-34a/b miR-193b miR-630	H2AX BRCA1 BRCA2 RAD51 p53	Human B lymphoblastic cell line IM9. Dose: 1; 10 Gy	MiR-34a may be involved in the cell cycle response and apoptosis pathway associated with p53.	[123,134,136,149,150]
miR-106a	ATM	Human prostate adenocarcinoma cell line PC3 Dose: 6 Gy	Promotion of cell survival and proliferation ability after irradiation.	[108]
miR-26b miR-107 miR-182 miR-155	ATM RAD51 BRCA1 RAD51	Human lymphoblast cell line TK6. Dose: 2 Gy	Regulation the cellular response to irradiation.	[26,104,133,151,152]
miR-106a miR-138 miR-193b	ATM H2AX BRACA1 BRCA2 RAD51	Human cell lines/head and neck (SCC-4, SCC-25, CAL-27) brain (LN229, T98G, U-87 MG). Dose: 2 Gy	MiRNAs induced significant changes in expression profiles.	[65,108,109,123]
